# Short-term air pollution and fracture admissions in Beijing

**DOI:** 10.3389/fpubh.2025.1644632

**Published:** 2025-09-03

**Authors:** Ning Wang, Rongrong Xu, Feng Luo, Renwei Cao, Zhongyu Wang, Shuo Chen, Xian Zhao, Shuai Lu, Yejun Zha, Yongjie Wei, Qiujin Xu, Minjuan Li

**Affiliations:** ^1^State Key Laboratory of Environmental Criteria and Risk Assessment, Chinese Research Academy of Environmental Sciences, Beijing, China; ^2^Center for Global Health, School of Public Health, Nanjing Medical University, Nanjing, China; ^3^College of Environmental Science and Engineering, Tongji University, Shanghai, China; ^4^Department of Orthopedic Trauma, National Center for Orthopaedics, Beijing Jishuitan Hospital, Capital Medical University, Beijing, China; ^5^Beijing Research Institute of Traumatology and Orthopaedics, Beijing, China

**Keywords:** air pollution, fracture, hospitalization, time-series study, lag effect

## Abstract

**Introduction:**

Limited evidence exists on the links between ambient air pollution and fractures. This study aimed to investigate the association between short-term exposure to criteria air pollutants and hospital admissions for fractures.

**Methods:**

We collected daily data on six criteria air pollutants and fracture admissions from Beijing Jishuitan Hospital between June 2021 and May 2023. Generalized additive models (GAM) with quasi-Poisson regression were applied to quantify the effects of air pollutants on admissions.

**Results:**

We identified 16,532 records on fracture admissions. At the lag03 day, an increase of 1 μg/m^3^ in PM_2.5_ and NO₂ was significantly associated with a 0.12% (95% CI: 0.01, 0.23%) and 0.26% (95% CI: 0.01, 0.51%) increase in fracture admissions, respectively. The exposure-response curve for PM_2.5_ showed a sharp rise after an initial decline at lower concentrations, which may be a model artifact. Stratified analyses revealed stronger associations in the younger population and a significant association between PM_2.5_ exposure and fracture risk in males. PM_2.5_ was also significantly associated with admissions for fragility fractures.

**Discussion:**

This study suggests that short-term exposure to air pollution, with observed cumulative lag effects, may increase the risk of fracture-related hospitalizations. These findings highlight the potential role of air pollutants as an environmental risk factor for fractures.

## 1 Introduction

Fracture, losses of bone integrity resulting from trauma, accidents, or comorbidities (1)—poses a global public health challenge as a leading cause of acute injury and chronic disability, with substantial socioeconomic burdens (2, 3). This burden is particularly pronounced among individuals with osteoporosis, a systemic bone disorder characterized by reduced bone density and increased fragility, which directly elevates fracture risk (4, 5). The Global Burden of Disease (GBD) Study highlights a marked rise in absolute fracture incidence between 1990 and 2019, with older adult populations demonstrating disproportionately higher vulnerability (6). Beyond individual health impacts, fractures impose multifaceted societal burdens, including work absenteeism, productivity loss, disability, diminished quality of life, and escalating healthcare costs (7–9). For instance, hip fractures often result in severe functional decline requiring prolonged rehabilitation and custodial care (10). The 2019 GBD assessment further underscores fractures as a critical component of global health loss metrics (11). While fracture etiology remains multifactorial, emerging evidence suggests air pollution may exacerbate fracture risk through bone density reduction and osteoporosis progression (12).

Air pollution constitutes a major global environment health threat (13), exemplified by China’s 2017 estimates of 124,000 air pollution-attributable deaths (95% UI: 108,000-140,000), including 85,000 from PM₂.₅ and 18,000 from ozone(O_3_) exposure (14). Although substantial evidence links long-term air pollution exposure to cardiopulmonary diseases, neurological impairments, and metabolic disorders (15–21), growing research highlights its broader systemic impacts, including potential associations with malignancies such as malignant brain tumors (22). However, the acute health impacts of short-term exposure—particularly on fracture risks—remain poorly characterized. Current mechanistic hypotheses suggest air pollutants may accelerate bone loss through oxidative stress and chronic inflammatory responses (23–27). Nevertheless, these proposed pathways primarily originate from chronic exposure studies yielding conflicting evidence: while multiple longitudinal investigations demonstrate PM₂.₅/NO₂-associated reductions in bone mineral density (BMD) (12, 28–32), other studies in populations with sustained traffic-related exposure report non-significant associations (33, 34).

To address this, the present study aims to investigate the association between short-term exposure to air pollutants (PM₂.₅, NO₂, etc.) and daily fracture hospitalizations in Beijing, quantify potential lag effects, and clarify whether such associations vary by demographic factors (sex, age) and fracture type. By doing so, we seek to fill the current knowledge gap regarding the acute musculoskeletal health impacts of air pollution.

Critically, this divergence underscores a critical gap: evidence regarding acute exposure impacts remains scarce, particularly in China, where recurrent short-term pollution events coincide with limited epidemiological data on musculoskeletal morbidity. To address this, this, the present study aims to investigate the association between short-term exposure to air pollutants (PM₂.₅, NO₂, etc.) and daily fracture hospitalizations in Beijing, quantify potential lag effects, and clarify whether such associations vary by demographic factors (sex, age) and fracture type. By doing so, we seek to fill the current knowledge gap regarding the acute musculoskeletal health impacts of air pollution.

## 2 Methods

### 2.1 Study population

This time-series study was conducted at Beijing Jishuitan Hospital, a tertiary orthopedic center. We extracted electronic medical records of hospitalized fracture patients from June 3, 2021, to May 25, 2023, using International Classification of Diseases-10 codes (ICD-10: S00-T14) through the hospital’s information system (HIS). To comprehensively capture fragility fractures (e.g., osteoporotic fractures), we retained cases with dual coding of traumatic fracture codes (S-codes) combined with M80.8, per WHO coding guidelines. Demographic and clinical data, including sex, age, and admission dates, were retrieved. To ensure geographical exposure consistency and diagnostic validity, we exclusively included patients with a household registration in Beijing and excluded outpatient/emergency cases. Only pathological fractures identified solely by ICD-10 codes M80-M84 without corresponding traumatic injury codes (e.g., malignant neoplasm-related M84.5, infection-related M84.6, or uncorrelated M80.8) and incomplete records were further removed to minimize confounding. The final sample comprised 16,532 cases, with a 5% random sampling validation confirming 98% data accuracy between source records and extracted variables. The study was approved by the Ethics Committee of Beijing Jishuitan Hospital (Approval No.: Ji Lun [K2024] No. [273]-00).

### 2.2 Meteorological and air pollutant data

Air pollution data were acquired from the National Urban Air Quality Real-time Release Platform (NUAQRRP) operated by the China National Environmental Monitoring Centre (CNEMC), which maintains a nationwide network of over 1,800 monitoring stations. Throughout the study period, mean concentrations of PM_2.5_, PM₁₀, NO₂, SO₂, and CO were calculated using 24-h average values, while O₃ levels were derived from the maximum 8-h moving average concentration recorded daily. City-wide daily averages were used as the primary exposure metric, since fracture inpatients resided across all 16 districts of Beijing, making this approach the optimal method to integrate city-scale pollution heterogeneity. The dataset demonstrated robust temporal completeness, with ≥90% data coverage per station, and spatial consistency verified via cross-validation between adjacent monitoring sites (PM_2.5_, R^2^ > 0.85; Supplementary Figure S1, S2).

Meteorological parameters, including daily mean temperature (°C) and dew-point temperature (°C), were retrieved from the Global Surface Summary of the Day (GSOD) database maintained by NOAA’s National Centers for Environmental Information.^1^ Representing urban-scale meteorological conditions, data were obtained from the World Meteorological Organization (WMO)-certified Beijing Capital International Airport Station (WBAN: 545110), ensuring standardized measurement protocols.

### 2.3 Statistical analyses

Daily admissions for fractures and air pollution concentrations were linked by date, allowing us to conduct a time-series analysis. We utilized a time-series methodology to conduct analysis, capitalizing on its inherent capacity to control for time-invariant confounders at the population level (35). Considering that the daily inpatient admissions for fractures approximated a quasi-Poisson distribution, we resorted to an over-dispersed generalized additive model (GAM) to explore the relationship between air pollution and daily inpatient admissions for fractures.

In our analysis, a natural spline function with 7 degrees of freedom (df) per year was applied to account for the long-term time trend. This choice aligns with standard practices in time-series studies on environmental health outcomes, as 7 df/year effectively captures long-term variations without overfitting, consistent with methods used in previous analyses of air pollution and health associations (36). Natural cubic smoothing functions were also employed to control the effects of daily temperature (6 dfs) and dew-point temperature (3 dfs). The 6 df for temperature was selected to address potential nonlinear relationships between temperature and fracture admissions, following approaches used in studies examining temperature-related health impacts (37). For dew-point temperature, 3 df was deemed adequate to adjust for humidity-related confounding while maintaining model parsimony, as excessive degrees of freedom could introduce noise into the analysis (35). For dew-point temperature, 3 df was deemed adequate to adjust for humidity-related confounding while maintaining model parsimony, as excessive degrees of freedom could introduce noise into the analysis. To evaluate the lagged impacts of air pollutants, we took into consideration both single-day lags (ranging from lag0 to lag4) and moving average lags (from lag01 to lag04). We further improved our model by incorporating an indicator for the day of week (DOW) and introducing a dummy variable to identify holidays. The main model is represented by the equation below:


$$\begin{array}{l} \text{Logit}[E(Y\_t)]=\alpha +\beta \times \text{Xpollutant}+\mathrm{ns}(\text{time},\mathrm{df}=7\times \text{year})\\ +\mathrm{ns}(\text{temperature},\mathrm{df}=6)+\mathrm{ns}(\mathrm{Dew}-\text{point temperature},\mathrm{df}=3)\\ +\mathrm{DOW}+\text{holiday} \end{array}$$

Where t represents the calendar time, and E(Yt) denotes the expected number of daily fracture inpatient admissions on day t. The term α is the intercept, and β is the regression coefficient (log odds ratio). Xpollutant refers to the specific pollutant, with ns indicating the natural spline function. Variables such as DOW and Holiday are included as dummy variables.

We reported the effect estimates and the corresponding 95% confidence intervals (CIs) as the percentage change in daily fracture inpatient admissions for every one-unit increase in each air pollutant. In addition to the single-pollutant models, we utilized a two-pollutant model to examine the independent association of air pollutants on daily fracture inpatient admissions. Although there were moderate to high correlations among individual pollutants, two-pollutant models were extensively used in sensitivity analyses in previous studies (36, 38, 39). To minimize collinearity, only pollutants with correlation coefficients below 0.7 were included in the model. We also plotted the exposure-response curves for the association between fracture inpatient admissions and ambient air pollution, using a natural spline function with 3dfs for air pollutants in the GAM model separately.

Furthermore, stratification analyses based on sex, age, and fracture type were carried out to explore potential effect modification. The age of inpatients with fractures was classified into two groups: those aged 0–64 years and those aged 65 years and over. The statistical significance of the differences in the effects of air pollution between the paired categories was determined by calculating the 95% CI as $(Q_{1}-Q_{2})\pm 1.96\sqrt{\;SE_{1}^{2}+SE_{2}^{2}\;}$, where *Q*_1_ and *Q*_2_ were the estimates of the effects in the paired categories, and *SE*_1_ and *SE*_2_ were their respective standard errors (39). We also calculated the *p* value according to the confidence intervals between subgroups (40).

We also conducted a sensitivity analyse to assess the robustness of the associations between air pollutants and fracture inpatient admissions. Firstly, previous studies have demonstrated potentially nonlinear and lagged associations between ambient temperature and adverse health outcomes (37). Hence, we re-analyzed the data by applying alternative degrees of freedom, ranging from 3 to 6, within the natural cubic spline framework for daily temperature.

In this study, all statistical tests were conducted using R software (Version 4.2.2), with the “mgcv” package for fitting GAMs. The statistical significance was evaluated on a two-sided basis, with a significance level set at 0.05.

## 3 Results

Table 1 summarizes the descriptive statistics of fracture hospitalizations, air pollutants and meteorological data during the study periods. A total of 16,532 fracture hospitalizations were recorded, of which 82.09% were identified as fragility fracture, and 17.91% as non-fragility fracture, with a daily mean of 40 visits. The males accounted for 33.20%, and the females accounted for 66.80%. 62.67% population were younger than 65 years.

**Table 1:** Summary statistics of daily fracture admissions, air pollution concentrations and weather conditions during the study period.

Variables	Total	Mean	SD	Min	*p* (25)	*p* (50)	*p* (75)	Max
Admissions	16,532	38.90	33.25	1	9	40	57	213
Sex								
Male	5,489	12.92	11.68	0	3	12	19	77
Female	11,043	25.98	22.16	0	7	27	38	136
Age								
0–64	10,360	24.38	21.38	0	6	24	36	140
Above 65	6,172	14.52	12.45	0	4	15	21	75
Fracture pattern								
Fragility fracture	13,570	31.93	26.52	0	8	33	47	162
Non-fragility fracture	2,962	6.969	7.47	0	1	6	10	51
Air pollution concentrations (24-h average, μg/m3)								
PM_2.5_ (ug/m^3^)		30.31	27.33	2.52	12.36	22.10	35.94	164.3
PM_10_ (ug/m^3^)		58.46	49.42	7.57	29.33	47.34	73.47	751.66
NO_2_ (ug/m^3^)		23.59	12.92	2.89	13.86	19.70	30.07	66.75
SO_2_ (ug/m^3^)		2.71	0.59	1.08	2.42	2.59	2.77	7.05
CO (mg/m^3^)		0.52	0.24	0.16	0.32	0.48	0.66	1.53
O_3_ (8-h) (ug/m^3^)		79.17	39.86	6.68	50.36	70.87	107.84	212.13
Meteorological measures (24-h average)								
Temperature (°C)		13.43	11.47	−12.72	3.75	14.33	24.17	30.72
Dew-point temperature (°C)		3.62	14.53	−27.83	−8.96	4.50	18.01	26.11

PM_2.5_, particles with aerodynamic diameter<2.5 μm; PM_10_, particles with aerodynamic diameter<10 μm; NO_2_, nitrogen dioxide; SO_2_, sulfur dioxide; CO, carbon monoxide; O_3_, ozone; SD, standard deviation.

During the study period, the median concentrations (medians, interquartile range [IQRs]) of air pollutants were 22.10 (12.36–35.94) μg/m^3^, 47.34(29.33–73.47) μg/m^3^, 19.70 (13.86–30.07) μg/m^3^, 2.59 (2.42–2.77) μg/m^3^, 0.48 (0.32–0.66) mg/m^3^, 70.87(50.36–107.83) μg/m^3^ for PM_2.5_, PM_10_, NO_2_, SO_2_, CO and O_3_. Mean daily temperature and dew point were 13.43 and 3.62 °C, respectively. Supplementary Table S1 presents the correlation coefficients among different air pollutants. There are strong positive correlations among PM_2.5_ PM₁₀, SO₂, NO₂, and CO, indicating that they potentially share common emission sources. O₃ shows negative correlations with NO₂ and PM₂.₅. There is a strong positive correlation between temperature and dew-point temperature. All pollutants except NO₂ are positively correlated with dew-point temperature (Supplementary Table S1).

**Figure 1:**
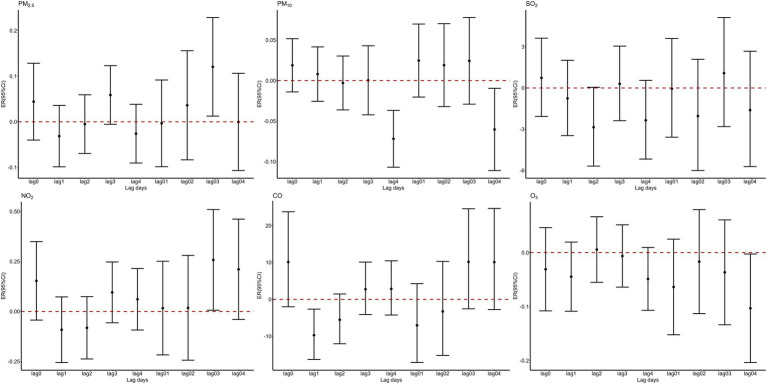
Percent changes of hospitalization volumes for fractures for a one-unit increase in air pollutants at different lag periods.

After adjusting for long-term trends, day-of-week variations, holidays, and meteorological factors, single-pollutant models were constructed for PM₂.₅, PM₁₀, NO₂, O₃, SO₂, and CO. Figure 1 illustrates the percentage change in fracture hospitalizations per 1 μg/m^3^ (1 mg/m^3^ for CO) increase in pollutant concentrations at different single-day lags (Lag0–Lag4) and moving average lags (Lag01–Lag04). Cumulative exposure analysis identified lag03 (0–3-day moving average) as the optimal exposure window for PM_2.5_ and NO₂, with statistically significant increases of 0.12% (95% CI: 0.01–0.23%) and 0.26% (0.01–0.51%) per 1 μg/m^3^ increment, respectively (*p* < 0.05). In contrast, SO₂ exhibited no significant associations across all lag structures.

**Figure 2:**
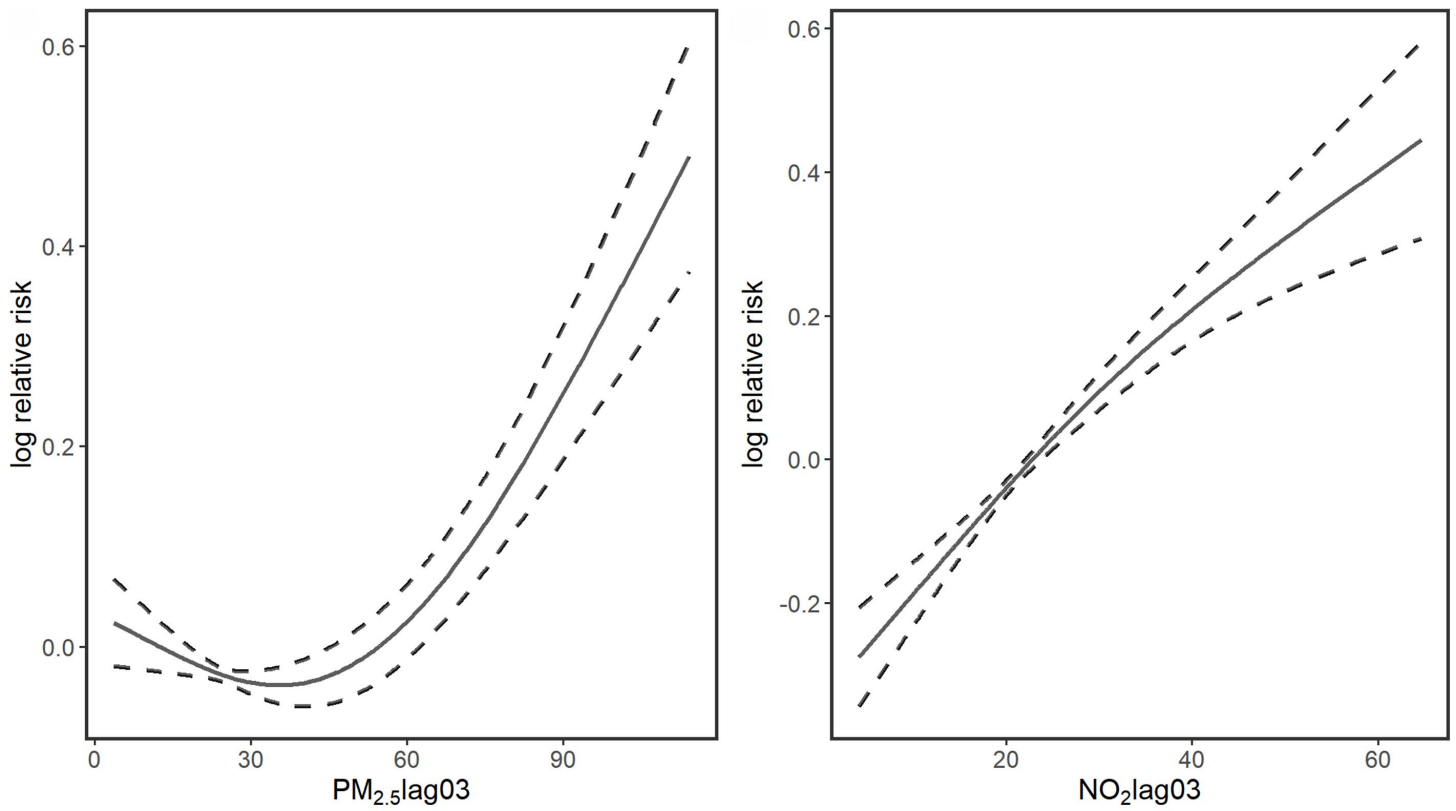
Smoothing plots of air pollutants (Lag 03) against fracture hospital admissions. X-axis is the pollutants concentrations (μg/m^3^). The solid lines indicate the estimated mean percentage of change in daily fracture hospital admissions, and the dotted lines represent 95% confidence interval.

Figure 2 presents the exposure-response curves for air pollutants (Lag 03) and fracture hospital admissions. The association of PM_2.5_ showed a pattern of a curve that decreased slightly at low concentrations and then increased sharply as the concentration rose. On the other hand, a near-linear upward trend was apparent for NO_2_. Even below the recommended daily levels in the Chinese Air Quality Standard (24-h average: 35 μg/m^3^ for PM_2.5_, 80 μg/m^3^ for NO_2_), we still observed significant associations of these air pollutants with fracture hospital admissions.

Based on the findings of the single—pollutant model, a stratified analysis was meticulously carried out, with a primary focus on PM_2.5_ and NO_2_, two atmospheric pollutants that exhibit notable associations with fracture outcomes. Table 2 comprehensively presents the impacts of these pollutants when stratified by gender, age, and fracture type. Regarding the cumulative 3-day lag of PM_2.5_ exposure, the study clearly demonstrates that a statistically significant association with fracture hospitalizations is observed solely among the male population (*p* < 0.05). Further in-depth analysis reveals that this association is more pronounced in the younger age group (0–64 years). Moreover, in comparison to non-fragility fractures, PM_2.5_ shows a more prominent correlation with fragility fractures. Conversely, for NO_2_, only negative results were obtained across all stratifications in terms of gender, age, and fracture type.

**Table 2:** Percent increase (mean and 95% confidence interval) in fracture hospitalization volumes associated with a per unit increase in air pollutant concentrations (moving average over 3 days, Lag 03), stratified by sex, age, and fracture pattern.

Variables	Total [Percent Increase (95% CI)]	Sex			Age (years)			Fracture pattern		
		Male [Percent Increase (95% CI)]	Female [Percent Increase (95% CI)]	*p*	0–64 [Percent Increase (95% CI)]	Above 65 [Percent Increase (95% CI)]	*p*	Fragility fracture [Percent Increase (95% CI)]	Non-fragility fracture [Percent Increase (95% CI)	*p*
PM_2.5_ lag03	0.12% (95%CI: 0.01–0.23%)	0.22% (95%CI: 0.03, 0.40)	0.08% (95%CI: −0.06-0.21%)	0.22	0.17% (95%CI: 0.04–0.30%)	0.03% (95%CI: −0.15-0.21%)	0.22	0.13% (95%CI: 0.01–0.25%)	0.09% (95%CI: −0.16-0.34%)	0.76
NO_2_ lag03	0.26% (95%CI: 0.01–0.51%)	0.34% (95%CI: −0.10-0.78%)	0.22% (95%CI: −0.09-0.53%)	0.66	0.38% (95%CI: 0.06–0.70%)	0.09% (95%CI: −0.32-0.50%)	0.27	0.20% (95%CI: −0.08-0.48%)	0.40% (95%CI: −0.22-1.01%)	0.58

The results of the two-pollutant models are shown in Table 3. In the two-pollutant model, when NO₂ is included in the model, the association between PM_2.5_ exposure and the number of fracture admissions becomes nonsignificant. However, this association still exists after adjusting for other pollutants. After adjusting for the exposure to other pollutants, the association between nitrogen dioxide and the number of fracture admissions turns out to be insignificant.

**Table 3:** Association between exposure to air pollutants and fracture hospitalization volumes in two-pollutant models.

Models	PM_2.5_	NO_2_
Unadjusted	0.12 (0.01–0.23)	0.26 (0.01–0.51)
Adjusted for PM_2.5_	—	0.14 (−0.16–0.45)
Adjusted for PM_10_	—	0.25 (−0.02–0.52)
Adjusted for NO_2_	0.08 (−0.05–0.22)	—
Adjusted for SO_2_	0.12 (0.01–0.23)	—
Adjusted for CO	—	0.21 (−0.09–0.52)
Adjusted for O_3_	0.12 (0.01–0.23)	0.25 (−0.01–0.50)

PM_2.5_, particles with aerodynamic diameter < 2.5 μm; PM_10_, particles with aerodynamic diameter < 10 μm; NO_2_, nitrogen dioxide; SO_2_, sulfur dioxide; CO, carbon monoxide; O_3_, ozone; SD, standard deviation. ^a^Pollutants with correlation coefficients below 0.7 were included in the model.

We performed multiple sensitivity analyses to check the robustness of our primary findings. The associations between air pollutants and the fracture admissions did not change substantially when using alternative degrees of freedom (dfs) for adjustment of temperature (Supplementary Table S2).

## 4 Discussion

Over a 3-year study period, an in-depth investigation was carried out on 16,532 fracture hospitalization cases at Jishuitan Hospital in Beijing, China. Notably, this study reveals that there is a significant relationship between the cumulative lag effects of short-term exposure to air pollutants including PM_2.5_ and NO_2_ and the increase in the number of fracture hospitalizations. Moreover, these associations are more prominent in the male population. Further analysis shows that the 0–64-year-old population is more sensitive to the impact of such air pollutants. It is particularly worth noting that there is a significant correlation between fragility fractures and PM_2.5_. To our knowledge, this study is one of the few studies focusing on the impact of ambient air pollution on the number of fracture hospitalizations.

Our study shows that there is a positive correlation between the number of fracture admissions and a series of air pollutants, such as PM_2.5_ and NO₂, which is consistent with the results of several previous studies (33). In a series of similar studies, the concentration of air pollutants, especially particulate matter, is negatively correlated with bone mineral density (33, 41–43). Chen et al. demonstrated that short-term exposure to PM_2.5_ led to an increase in the number of outpatient visits for knee osteoarthritis in Beijing, suggesting the possible existence of additional air pollution-related fractures. A study by Prada et al. (44) analyzed 9.2 million medical records and found an association between the mass concentration of PM_2.5_ and the fracture admission rate. For every 4.18 μg/m^3^ increase in PM_2.5_, the hospitalization rate for fractures among the older adults increased by 4.1%. A study in southern Europe analyzed the short-term effects of different outdoor air pollutants (SO₂, NO, NO₂, O₃) and suspended particulate matter (PM_2.5_, PM₁₀) on the incidence of osteoporotic fractures and found an association between the incidence of hip fractures and nitrogen dioxide (NO₂) (incidence rate ratio was 1.02 (95% confidence interval was 1.01–1.04)). For ozone (O₃) levels, this association was negative (incidence rate ratio was 0.97 (95% confidence interval was 0.95–0.99)) (45). In a retrospective cohort study (46), it was shown that an increase in exposure to CO and NO₂ would increase the risk of osteoporosis. Nevertheless, a study showed that exposure to NO₂ was not significantly associated with an increased risk of fracture hospitalizations (47). The differences in the associations observed in the studies may be attributed to regional differences in study design, population characteristics, and air pollution levels.

This study reveals the age- and gender-specific effects of PM_2.5_ and NO_2_ on the number of hospital admissions in lag03. In the age-stratified analysis, we found that the exposure effects of air pollutants were more significant in the 0–64-year-old population, which is different from the conclusion of Gu et al.’s national time (48)—series analysis. Their research showed that the association between PM_2.5_ and musculoskeletal system diseases was stronger in the 65–74-year-old and ≥75-year-old groups. This discrepancy can likely be attributed to the combined effects of distinct exposure patterns and behavioral factors. On one hand, in terms of exposure, industries such as construction and transportation in Beijing employ a significant young workforce, leading to inevitably higher levels of outdoor occupational exposure. On the other hand, from a behavioral perspective, younger populations are also more inclined to engage in risk-taking activities while commuting in polluted conditions, thereby further elevating their health risks. Notably, this study found consistency with the results of five independent cohort studies (47, 49–52), all of which reported that the risk of osteoporosis in the middle-aged and young people (> 40-year-old, 50–59-year-old, < 65-year-old, 50–74-year-old, < 60-year-old) was significantly higher than that in the adults aged 65 and older. In the gender-stratified analysis, the data showed that the impact of air pollutants on the fracture risk in men was significantly stronger than that in women, and the effect of PM_2.5_ on the fracture risk in men was significant. This finding is consistent with the research conclusion of Sun et al. (52) and is supported by the evidence of increased osteoporosis risk in men in three cohort studies (50, 52, 53). Regarding the gender differences, existing evidence points to a pathway centered on endocrine disruption. Some studies have shown that the relationship between the exposure concentration of PM_2.5_ and the change of serum testosterone level is the closest. Whether it is the cumulative effect or the single-day lag effect, long-term exposure to PM_2.5_ can reduce the serum testosterone level in men (for every 10 μg/m^3^ increase in PM_2.5_, testosterone decreases by 4.2%), thus weakening the ability to maintain bone density (54). Therefore, the guidelines of the Endocrine Society recommend bone density measurement for men with hypogonadism (54), because hypogonadism can lead to a decrease in bone density (55).

In addition, animal experiments (56) have shown that the exposure to PM_2.5_ combined with tobacco smoke can significantly inhibit the differentiation of osteoblasts and exacerbate bone loss in male mice. Considering the substantially higher smoking prevalence among men (36.6%) compared to women (2.2%) in Beijing (2022), this pollutant-lifestyle interaction may represent a significant factor contributing to the elevated fracture risk observed in males.

Based on the detailed classification of injury mechanisms in medical records, this study divided fracture cases into two major categories: fragility fractures and non-fragility fractures. Among them, fragility fractures specifically refer to hip, vertebral and distal radius fractures caused by low-energy trauma (such as a fall from standing height), while non-fragility fractures are defined as traumatic fractures caused by high-impact injuries (such as traffic accidents). Data analysis showed that fragility fractures accounted for 82.09% of all cases and were positively correlated with PM_2.5_ exposure (*p* < 0.05), which is highly consistent with previous mechanism studies on air pollution affecting bone health by interfering with bone metabolism pathways (57, 58). Notably, although non-fragility fractures accounted for 17.91% and did not reach statistical significance (*p* ≥ 0.05), a positive correlation was also shown.

The mechanism underlying the increase in fracture admissions associated with elevated atmospheric pollutant concentrations remains unclear. If reduced bone resistance were the causal pathway linking air pollution to fractures, such an effect would not be expected to manifest over short timeframes. Therefore, we propose that the observed short-term association is more plausibly explained by air pollution increasing fracture risk through acute traumatic events. A more direct hypothesis suggests that pollutants elevate fracture admissions in the short term by raising the incidence of acute incidents such as falls and traffic accidents (45). Crucially, our findings—where fragility fractures (predominantly caused by falls) constituted the majority of cases and showed significant associations with pollution—provide compelling epidemiological support for this “fall hypothesis.” Falling is one of the most common health problems faced by older adults nowadays (59). Most falls are not caused by a single reason, but are the result of the interaction between personal factors and environmental factors (45). The internal characteristics of an individual may make them prone to falling, but environmental factors or acute illnesses are often important causes of falls. Changes in blood pressure may be one of the reasons for falls. Some studies have shown that short-term exposure to atmospheric pollutants may cause arrhythmia and changes in blood pressure (60, 61), which can lead to falls and further increase the risk of fractures (62). One of the biological mechanisms by which several pollutants have a short-term impact on the risk of cardiovascular events is the impairment of the autonomic nervous system (ANS) response (60, 61). Under normal circumstances, the rhythmic activity of the heart is controlled by the autorhythmic cells in the sinoatrial node, which is in turn regulated by the vagus nerve. Acute exposure to PM_2.5_ may stimulate the autonomic nervous system, protemtially increasing the risk of arrhythmia, orthostatic hypotension, and fainting (60). In addition, pollutants may lead to high-impact trauma events, such as traffic accidents. Air pollution may reduce the driving performance of drivers, thus leading to traffic accidents (63). However, this impact has been almost ignored in the literature. Shi et al. investigated the short-term impact of air pollutants on the number of traffic fatalities based on the daily urban panel data in China from 2013 to 2018. They pointed out that for every 1 μg/m^3^ increase in the concentration of PM_2.5_, the number of traffic fatalities increased by 0.64%. A multicenter cross-sectional study (64) conducted in Taiwan in 2018 showed that an unhealthy air quality index, exposure to high concentrations of fine particulate matter, etc. were risk factors for severe injuries, while ozone was negatively correlated with the risk of suffering severe injuries. The negative impact of air pollutants on traffic safety can be considered from two aspects. Firstly, air pollution may reduce transportation efficiency, thus causing traffic congestion and affecting traffic safety. Secondly, air pollution may affect the physiological state and cognitive ability of drivers, thus influencing the driving state of drivers and ultimately leading to traffic accidents (65–67).

Current research on the association between air pollutants and fractures has primarily focused on European and American countries. This study, based on fracture admission data from a tertiary hospital in Beijing, China, investigated the short-term effects of air pollutants on fracture hospitalization rates. This study has several limitations that should be noted. First, as an ecological study, it uses average outdoor pollutant concentrations as a proxy for individual exposure levels, which may introduce exposure measurement bias. Second, the single-hospital design in Beijing could limit geographic generalizability. However, the selected hospital is a large tertiary care center with specialized orthopedic services, annually admitting a high volume of fracture patients. The large sample size and high-quality clinical data partially mitigate potential selection bias inherent in single-center studies. Third, this study exclusively analyzed hospitalized fracture cases. While this approach offers the advantage of more comprehensive clinical information—such as injury mechanisms and diagnostic details—enabling accurate classification, it also limits the generalizability of the results to this specific patient population.

## 5 Conclusion

In conclusion, short-term exposure to air pollutants (particularly PM₂.₅ and NO₂) is associated with an increased risk of fracture admissions in Beijing, with evidence of cumulative lag effects. These findings contribute to the growing body of evidence linking ambient air pollution to acute musculoskeletal health outcomes, highlighting the need for further research on potential biological mechanisms and targeted public health strategies to mitigate such risks.

## Data Availability

The raw data supporting the conclusions of this article will be made available by the authors, without undue reservation.

## Footnotes

^1^ https://www.ncei.noaa.gov/data/global-summary-of-the-day/archive/

## Supplementary material

Supplementary Figure 1 Distribution of Xinjiekou Hospital and Air Quality Monitoring Stations.

Supplementary Figure 2 Distance Statistics Between Xinjiekou Hospital and Monitoring Stations.
