# Supplementary figures and images for: Short-term air pollution and fracture admissions in Beijing
Source: Front Public Health. 2025 Sep 3;13:1644632. doi: 10.3389/fpubh.2025.1644632 (PMC12441165; doi:10.3389/fpubh.2025.1644632)

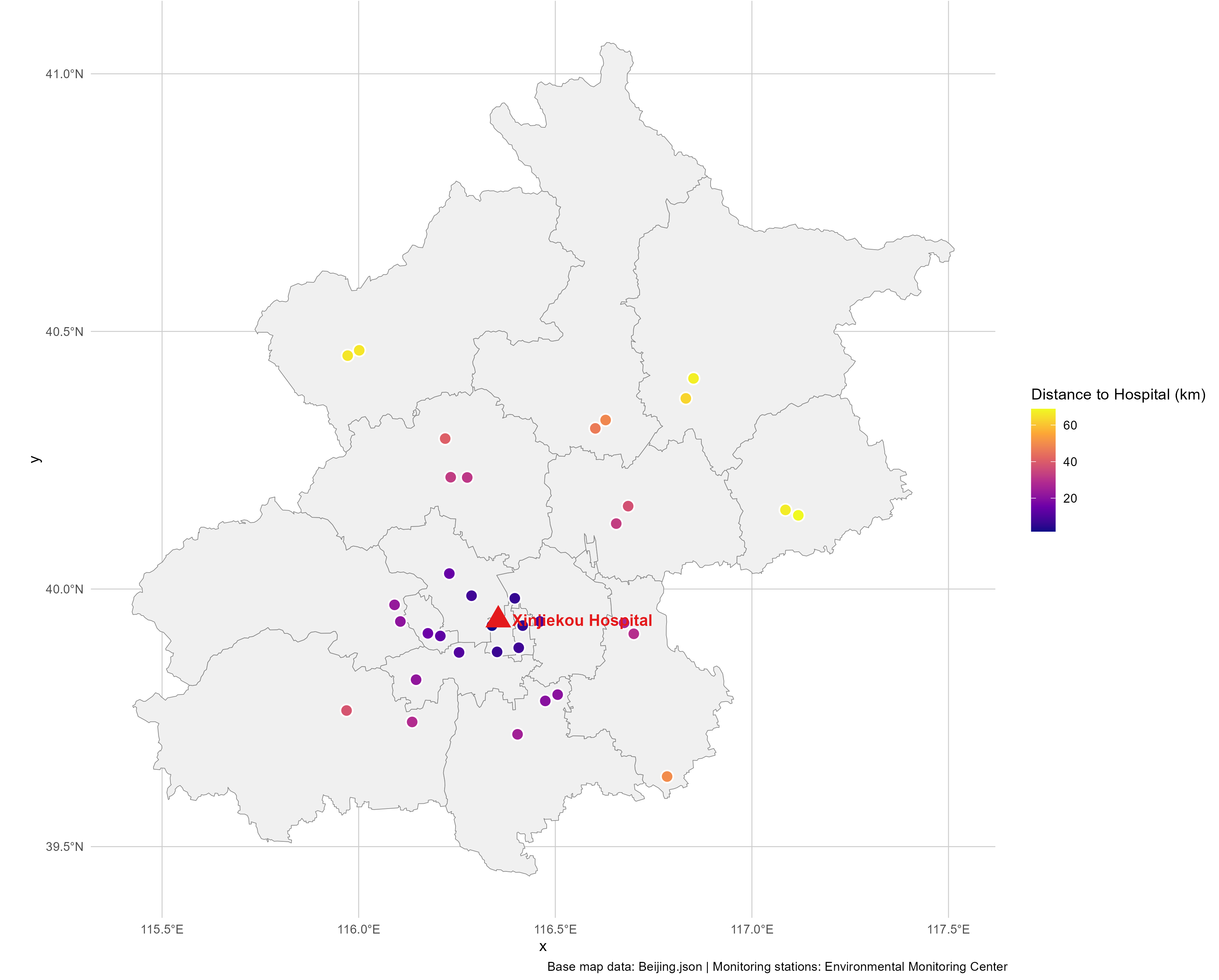

Supplement: Supplementary Figure 1 — Distribution of Xinjiekou Hospital and Air Quality Monitoring Stations. [file Image_1.tif]

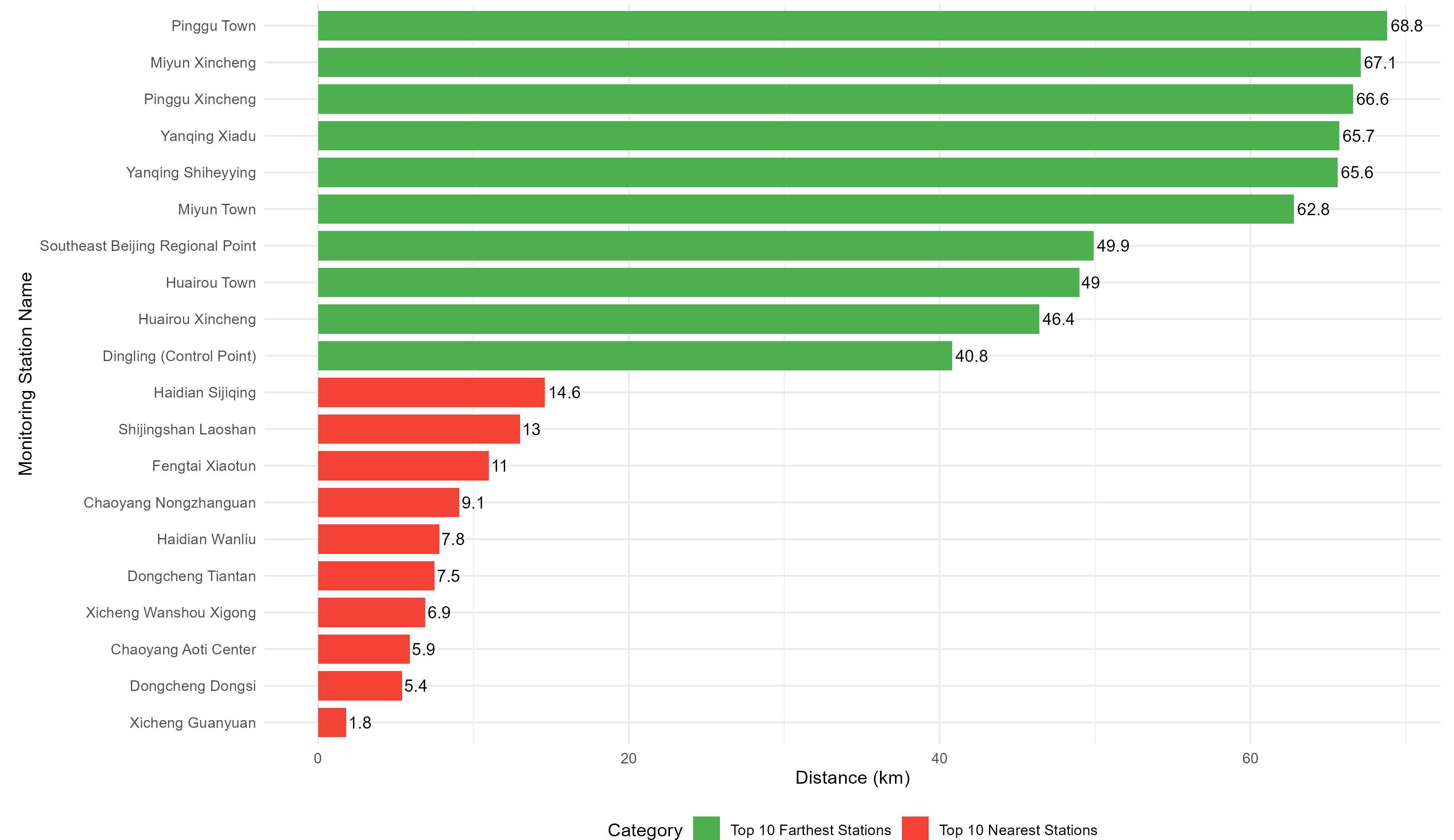

Supplement: Supplementary Figure 2 — Distance Statistics Between Xinjiekou Hospital and Monitoring Stations. [file Image_2.tif]
