# Supplementary material for: Short-term air pollution and fracture admissions in Beijing
Source: Front Public Health. 2025 Sep 3;13:1644632. doi: 10.3389/fpubh.2025.1644632 (PMC12441165; doi:10.3389/fpubh.2025.1644632)
Supplement: Supplementary file 3 [file Table_1.docx]

| **Supplementary Table 1. Spearman correlation coefficients between daily air pollutant concentrations and weather conditions during the study period** | | | | | | | |
| --- | --- | --- | --- | --- | --- | --- | --- |
|  | PM_10_ | SO_2_ | NO_2_ | CO | O_3_ | Temp | DEWP |
| PM_2.5_ | 0.896** | 0.315** | 0.662** | 0.764** | -0.026 | 0.003 | 0.132 |
| PM_10_ |  | 0.283 | 0.644** | 0.576** | 0.004** | -0.018** | 0.011** |
| SO_2_ |  |  | 0.207** | 0.395** | 0.149** | 0.258** | 0.306** |
| NO_2_ |  |  |  | 0.471** | -0.416** | -0.310** | -0.214** |
| CO |  |  |  |  | 0.019** | 0.225** | 0.450** |
| O_3_ |  |  |  |  |  | 0.764** | 0.610** |
| Temp |  |  |  |  |  |  | 0.909** |
| Abbreviations: PM_2.5_, particles with aerodynamic diameter<2.5 μm; PM_10_, particles with aerodynamic diameter<10 μm; NO_2_, nitrogen dioxide; SO_2_, sulfur dioxide; CO, carbon monoxide; O_3_, ozone; Temp, temperature; DEWP, Dew-point temperature.  ** p-value < 0.001 | | | | | | | |

| **Supplementary Table 2. Percent change (means and 95% confidence intervals) in fracture hospitalization volumes associated with a one-unit increase in air pollutants concentrations (lag03) by different degrees of freedoms for the temperature cubic splines.** | | | | |
| --- | --- | --- | --- | --- |
| ***Dfs*** | **3** | **4** | **5** | **6** |
| PM_2.5_ | **0.13 (0.02-0.24)** | **0.13 (0.02-0.24)** | **0.13(0.02-0.24)** | **0.12(0.01-0.23)** |
| NO_2_ | **0.27(0.02-0.52)** | **0.27(0.02-0.52)** | **0.27(0.02-0.52)** | **0.26( 0.01-0.51)** |
| Abbreviations: Abbreviations: df, degree of freedom  Notes: ^a^Pollutant concentrations represent 3-day moving averages (Lag 03).  ^b^Associations with statistical significance are highlighted in bold. | | | | |
